# The International Congress

**Published:** 1887-08

**Authors:** 


### THE INTERNATIONAL CONGRESS

To convene at Washington in September next, is a contemplated event of great importance.

We have on a former occasion expressed our conviction to this effect, and our hope that the profession throughout the Union will lay aside all prejudices and petty jealousies growing out of local differences, whatever these may be; or whatever supposed irregularities or seemingly unfair discriminations in the matter of appointments may have occurred in the organization of the Congress. The honor of the medical profession, the national pride and patriotism of our people and the great interests of medical science throughout the world demand that the Congress should be sustained. The very small appropriation made by the United States Congress in behalf of this important object is a matter of great regret and mortification to the medical profession.

Men of advanced and enlarged views cannot but feel surprised at this indifference and evident lack of appreciation of the obligations of the public to the medical profession.

When we look back upon the vast benefits which have accrued to mankind from the discoveries in medical science, it is amazing that there should exist in this progressive country so great indifference to these facts and so great aversion on the part of our statesmen to extending the benefits of a liberal legislation to the promotion of medical science.

Who can estimate the immense value to mankind which has flowed from the discovery of vaccination, from ænesthetic agents, from vivisections, microscopic investigations, surgical appliances, etc? And these investigations, led by the labors of the self sacrificing devotees of medical science, still go on in the face of all this indifference by the public, and despite the niggardly policy of the legislators of a great and wealthy government.

In the present instance the leading members of the profession from the whole world are to meet in an International Congress at Washington City, to consult upon and discuss great measures looking to the public health, and to the advancement of all that pertains to medical science; and yet when Congress was called upon to encourage and aid in this great work, the great minds in that body seemed almost wholly indifferent to the fact that a congress of the great minds in the profession from the whole world has been called to Washington designed to discuss the highest and grandest interests of science and of humanity.

So small was the appreciation of the great objects involved, and so blind to our national fame and pride that only a meagre and insufficient appropriation was made in its behalf, and the profession everywhere are called upon to contribute from their own pockets to meet the expenses of the Congress and to save the honor of the profession and the American government from the shame and disgrace which would result from a failure of the Congress.

We are glad to learn that certain of the medical societies and the members of the profession are rallying to the support of the Medical Congress, and that many medical men of Atlanta are aiding in the laudable and patriotic work. Push on the ball.

